# Influences of Reservoir Conditions on the Performance of Cellulose Nanofiber/Laponite-Reinforced Supramolecular Polymer Gel-Based Lost Circulation Materials

**DOI:** 10.3390/gels11070472

**Published:** 2025-06-20

**Authors:** Liyao Dai, Jinsheng Sun, Kaihe Lv, Yingrui Bai, Jianlong Wang, Chaozheng Liu, Mei-Chun Li

**Affiliations:** 1 School of Petroleum Engineering, China University of Petroleum (East China), Qingdao 266580, China; b22020047@s.upc.edu.cn (L.D.); sunjsdri@cnpc.com.cn (J.S.); lkh54321@126.com (K.L.); smartbyron@upc.edu.cn (Y.B.); 2 State Key Laboratory of Deep Oil and Gas, China University of Petroleum (East China), Qingdao 266580, China; 3 CNPC Engineering Technology R&D Company Limited, Beijing 102206, China; wjldr@cnpc.com.cn; 4 Co-Innovation Center of Efficient Processing and Utilization of Forest Resources, College of Materials Science and Engineering, Nanjing Forestry University, Nanjing 210037, China; lczwood@163.com; 5 Shandong Key Laboratory of Oil and Gas Field Chemistry, China University of Petroleum (East China), Qingdao 266580, China

**Keywords:** lost circulation, supramolecular polymer gel, reservoir conditions, influence factor

## Abstract

Lost circulation during drilling has significantly hindered the safe and efficient development of oil and gas resources. Supramolecular polymer gel–based lost circulation materials have shown significant potential for application due to their unique molecular structures and superior performance. Herein, a high–performance supramolecular polymer gel was developed, and the influence of reservoir conditions on the performance of the supramolecular polymer gel was investigated in detail. The results identified an optimal formulation for the preparation of supramolecular polymer gel comprising 15 wt% acrylamide, 3 wt% 2-acrylamide-2-methylpropanesulfonic acid, 2.6 wt% divinylbenzene, 5 wt% polyvinyl alcohol, 0.30 wt% cellulose nanofibers, and 3 wt% laponite. The performance of the gel-forming suspension and the resulting supramolecular polymer gel was influenced by various factors, including temperature, density, pH, and the intrusion of drilling fluid, saltwater, and crude oil. Nevertheless, the supramolecular polymer gels consistently exhibited high strength under diverse environmental conditions, as confirmed by rheological measurements. Moreover, the gels exhibited strong plugging performance across various fracture widths and in permeable formations, with maximum breakthrough pressures exceeding 6 MPa. These findings establish a theoretical foundation and practical approach for the field application of supramolecular polymer gels in complex geological formations, demonstrating their effectiveness in controlling lost circulation under challenging downhole conditions.

## 1. Introduction

In drilling engineering, drilling fluid plays a vital role by transporting rock cuttings to the surface, maintaining borehole cleanliness, and balancing formation pressure through hydrostatic pressure to prevent blowouts [1,2,3,4]. However, due to pressure differentials and the complex pore and fracture structures of the formation, downhole fluids (e.g., drilling and completion fluids) are prone to invading the formation or allowing formation fluids to flow back in. This can lead to significant drilling fluid losses and disrupt the pressure balance. When drilling fluid extensively leaks into the formation, the phenomenon is referred to as lost circulation. Lost circulation can result in substantial fluid loss, increased operational costs, wellbore instability (e.g., borehole enlargement or collapse), and reservoir damage that reduces production capacity, thereby hindering the safe and efficient development of oil and gas resources [5,6].

As oil and gas exploration and development extend into more geologically complex formations, reservoir conditions are becoming increasingly variable. These complex conditions impose higher performance requirements on lost circulation materials. Traditional lost circulation materials often suffer from poor plugging performance and limited adaptability, making them inadequate for complex reservoirs. Supramolecular polymer gels, as a new class of lost circulation materials, show great application potential due to their unique molecular structures and outstanding performance characteristics [7,8,9]. The precursory monomers can react in situ within leakage channels with tunable gelling time, forming a mesh-like structure with sufficient strength via non-covalent interactions, thereby effectively sealing the leakage paths. Compared to conventional materials, supramolecular polymer gels exhibit superior sealing strength and adaptability in complex formations.

In recent years, significant progress has been made in research on supramolecular polymer gel-based lost circulation materials. Sun et al. [10] designed a supramolecular polymer gel by copolymerizing polydopamine, the cationic monomer (3-acrylamidopropyl)trimethylammonium chloride (APTAC), and acrylamide. This gel was capable of sealing 1 mm fractures and withstanding a breakthrough pressure of 7.6 MPa at 90 °C. The adhesive properties of polydopamine, combined with the electrostatic interactions of APTAC’s cationic groups, enhanced the gel’s interfacial sealing efficiency under moderate-temperature and small-fracture conditions. However, in high-temperature environments, the performance of this gel system may be compromised due to potential degradation in strength and sealing ability. To address these limitations, Yang et al. [11] developed downhole cross-linked supramolecular polymer gels via a one-pot synthesis using acrylamide and octadecyl methacrylate as monomers. Owing to the synergistic effects of hydrophobic association and hydrogen bonding, this gel retained high strength (G′ > 0.16 MPa) after aging at 135 °C, thereby expanding the applicable temperature range of downhole gel materials.

To further enhance the performance of supramolecular polymer gels, a wide range of nanomaterial-based additives have been applied [8,12,13,14,15,16,17,18,19,20,21,22,23,24,25,26]. For instance, in our previous work, cellulose nanofibers (CNFs) were successfully employed to enhance the overall performance of supramolecular polymer gels [8]. CNFs, derived from renewable biomass, possess high aspect ratios, excellent mechanical strength, and a network-forming ability due to abundant hydroxyl groups [27,28,29]. When incorporated into polymer gels, CNFs reinforced the gel matrix through hydrogen bonding and entanglement, improving its mechanical strength, elasticity, and resistance to shear deformation. This enhanced the gel’s ability to withstand high-pressure environments and maintain stability under downhole conditions.

On the other hand, laponite, a synthetic layered silicate with nanoscale disc-like morphology, contributes to the thermal stability and rheological performance of the gel [30]. Due to its high surface area and strong electrostatic interactions, laponite can form a thixotropic network within the gel, improving the gel’s ability to resist fluid invasion by sealing microfractures and enhancing the overall integrity of the gel structure [31,32,33]. Therefore, the synergistic use of CNFs and laponite in supramolecular polymer gels is expected to result in improved gelation behavior, enhanced structural strength, and better adaptability to complex formation conditions. These enhancements are crucial for ensuring the long-term effectiveness of the gel in preventing lost circulation in high-temperature and high-salinity reservoirs. However, the actual reservoir environments are highly heterogeneous. Variations in temperature, pressure, fluid density, salinity, and other parameters across different formations can directly or indirectly affect the gelation process and plugging performance of supramolecular polymer gels.

This study aims to systematically investigate the impact of complex reservoir conditions on the performance of CNF/laponite-reinforced supramolecular polymer gel-based lost circulation materials. A base gel formulation was first developed using acrylamide (AM), 2-acrylamido-2-methylpropanesulfonic acid (AMPS), and polyvinyl alcohol (PVA) as primary components. The concentrations of CNFs and laponite were then optimized to enhance the supramolecular polymer gel’s performance. The resulting supramolecular polymer gels were then evaluated under representative reservoir conditions, including variations in temperature, fluid density, and salinity, to assess their effects on gelation time, rheological behavior, thermal stability, and plugging efficiency. This work is expected to elucidate the fundamental relationships between reservoir parameters and gel performance, providing a solid theoretical and technical basis for the application of supramolecular polymer gels in complex formations.

## 2. Results and Discussion

### 2.1. Preparation and Optimization of Supramolecular Polymer Gels

Supramolecular polymer gels were synthesized via free radical polymerization, with a base formulation comprising a two-component network structure. The first component is a PVA-based network, in which the abundant hydroxyl groups act as active sites for intramolecular and intermolecular hydrogen bonding [34]. The second component is a copolymer matrix consisting of AM, AMPS, and DVB. The vinyl and amide groups in AM exhibit high reactivity and hydrogen bonding capabilities [35]. The sulfonic acid and amide groups in AMPS synergistically enhance water solubility and salt resistance, while the aromatic ring and divinyl functional groups in DVB contribute to increased thermal stability and crosslinking density [36].

Given the synergistic effects of these four monomers in enhancing gel mechanical properties, an L9 (3^4^) orthogonal experimental design was employed to systematically study the influence of four factors: Factor A (AM), Factor B (AMPS), Factor C (DVB), and Factor D (PVA), each at three levels, on gel strength. The experimental design parameters are summarized in Table 1.

**Figure 1:**
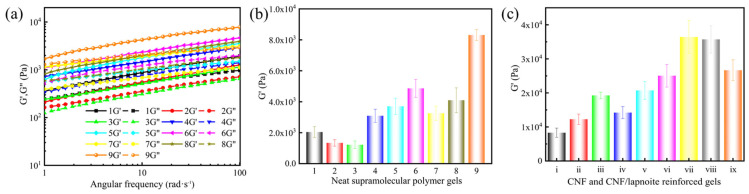
Viscoelasticity of supramolecular polymer gels: (**a**) dynamic frequency sweep at a fixed strain of 10%, (**b**) storage modulus at a fixed frequency of 84 rad/s, and (**c**) storage modulus after adding CNFs and CNF/laponite at a fixed frequency of 84 rad/s (i—neat, ii—0.15 wt% CNFs, iii—0.30 wt% CNFs, iv—0.45 wt% CNFs, v—0.30 wt% CNFs + 2.0 wt% LAP, vi—0.30 wt% CNFs + 2.5 wt% LAP, vii—0.30 wt% CNFs + 3.0 wt% LAP, viii—0.30 wt% CNFs + 3.5 wt% LAP, ix—0.30 wt% CNFs + 4.0 wt% LAP).

Based on the orthogonal experimental design, nine gel formulations were selected (Table 2), and their viscoelastic properties were evaluated, including the storage modulus (G′) and loss modulus (G″) (Figure 1a). Notably, G′ is a key indicator of a gel’s elastic behavior and structural integrity. A higher G′ value reflects a stronger, more rigid network capable of resisting deformation under stress. In lost circulation control, gels with high G′ more effectively seal fractures, maintain their shape under pressure, and resist extrusion by various fluids. Furthermore, increased G′ improves the gel’s mechanical strength and pressure-bearing capacity, which is essential for reliable performance in complex reservoir conditions. Therefore, optimizing G′ through formulation adjustments ensures the gel remains stable, durable, and effective as a high-performance lost circulation material. Range analysis of the orthogonal experiment data (Figure 1b) revealed the influence of each factor on gel strength in the order: A (AM) > B (AMPS) > D (PVA) > C (DVB). The optimal formulation was determined to be AM (A_3_, 15 wt%), AMPS (B_3_, 3 wt%), DVB (C_2_, 2.6 wt%), and PVA (D_1_, 5 wt%), which produced a gel with the highest G′ of 8313 Pa.

To further enhance the gel strength of supramolecular polymer gels, CNFs and laponite were incorporated into the network structure. Among them, CNFs exhibit their fibrous structure, with diameters ranging from 1 to 100 nm and lengths up to several micrometers, and have very high specific surface areas and aspect ratios. Additionally, their surfaces contain abundant hydroxyl groups, ensuring good hydrophilicity and compatibility. Laponite, in contrast, exhibits a 2:1 layered crystal structure with strong electronegativity due to its inherent negative charges, making it strongly electronegative. At certain concentrations, laponite forms a stable three-dimensional network structure [8,37]. Concentration-gradient experiments (Figure 1c) demonstrated that both nanomaterials enhanced gel strength in a concentration-dependent manner. When CNFs were added at 0.30 wt%, the G′ increased by 232%, attributed to their uniform dispersion and strong hydrogen bonding with the polymer chains. Building on the optimized 0.30 wt% CNFs, the subsequent addition of laponite further improved the gel strength, reaching a maximum G′ at 3 wt%. This enhancement was primarily due to the formation of a more robust three-dimensional network through hydrogen bonding and electrostatic interactions. However, excessive addition of either additive led to the aggregation of CNFs and laponite, which compromised the gel structure. Therefore, the optimal concentrations were determined to be 0.30 wt% CNFs and 3 wt% laponite.

### 2.2. Influence of Reservoir Conditions on the Performance of Supramolecular Polymer Gels

In complex reservoir environments, conditions such as temperature, pressure, pH, and the intrusion of different wellbore fluids (i.e., drilling fluids, saltwater, and crude oil) significantly influence the performance of supramolecular polymer gels. High temperatures can disrupt hydrogen bonding and physical crosslinks in supramolecular polymer gels, potentially weakening their structure and reducing gel strength. Similarly, high formation pressure necessitates the use of high-density gel-forming suspensions, which may affect gelation kinetics and increase the risk of premature syneresis or phase separation. Extreme pH conditions affect the ionic interactions within the gel network, impacting gel stability and performance. Additionally, the intrusion of different wellbore fluids can interfere with the gel’s formation by introducing contaminants that disrupt the chemical balance, affecting the gel’s consistency and strength. Therefore, it is essential to investigate how temperature, density, pH, and intrusion of drilling fluid, saltwater, and crude oil impact the gel strength of supramolecular polymer gels to ensure their reliability and effectiveness under different reservoir conditions.

#### 2.2.1. Temperature

Formation temperature is a critical factor affecting both the gelation process and the final properties of supramolecular polymer gels. Herein, the supramolecular polymer gels were prepared under four different temperatures: 80 °C, 100 °C, 120 °C, and 140 °C, followed by an evaluation of their viscoelastic properties.

**Figure 2:**
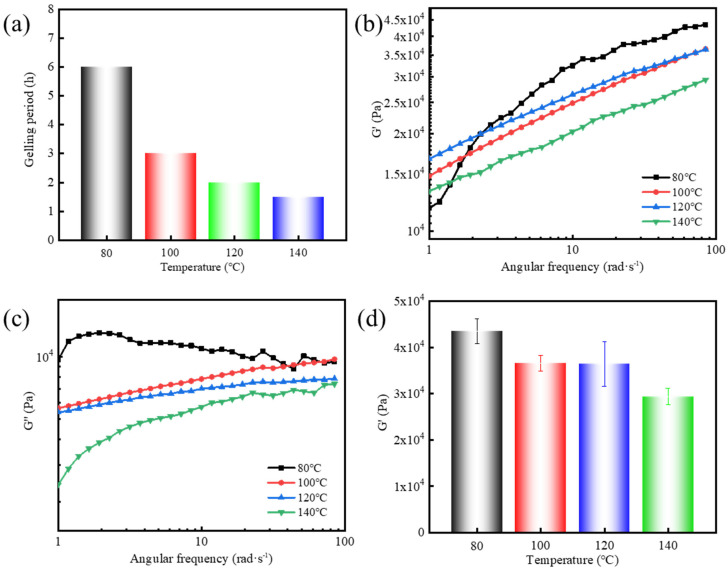
Effect of temperature on the gelation process and viscoelasticity of supramolecular polymer gels: (**a**) gelation time; (**b**) G′ versus angular frequency, (**c**) G″ versus angular frequency, and (**d**) G′ at a fixed angular frequency of 84 rad/s.

Figure 2a shows the variation in gelation time with temperature for the supramolecular polymer gels. It can be observed that the gelation time of the supramolecular polymer gels exhibited a negative correlation with temperature. Specifically, as the temperature increased from 80 °C to 140 °C, the gelation time decreased from 6 h to 1.5 h. The acceleration of gelation is primarily attributed to two factors: (1) the faster melting of the resin matrix on the surface of the resin-coated initiator at higher temperatures, and (2) the reduced half-life of the initiator with increasing temperature. The reduced gelation time is significant because faster gelation allows for more efficient application in field operations, where time is critical. Shorter gelation times may also be beneficial for applications requiring quick sealing. However, this also presents challenges, as rapid gel formation may result in an incomplete or less dense gel structure, potentially weakening its sealing ability. Therefore, understanding the effect of temperature on gelation time is essential for optimizing gel formulations for specific reservoir conditions while balancing the need for timely gel formation and structural integrity.

To confirm our assumption, the viscoelasticity of the resulting supramolecular polymer gels was evaluated (Figure 2b–d). It can be seen that the G′ gradually decreased with an increase in the temperature (Figure 2d). The highest G′, 43,459 Pa, was observed at 80 °C, while it decreased to 29,361 Pa at 140 °C. As discussed previously, this decline may result from rapid gelation that reduces the stiffness of the network. Additionally, high temperatures weaken non-covalent interactions (e.g., hydrogen bonding and electrostatic forces), thereby reducing the overall gel strength [38].

#### 2.2.2. Density

Having established the pronounced influence of temperature on the rheological properties of supramolecular polymer gels, we now investigate how variations in density may further modulate their performance under reservoir conditions. Due to pressure differences across various formations, the density of the gel-forming suspension must be carefully tailored. Barite is commonly used as a weighting material to increase the density of drilling fluids and balance formation pressure [39]. Therefore, in this study, different amounts of barite were added to gel-forming suspensions to achieve various densities (1.00, 1.05, 1.10, 1.15, 1.20, and 1.25 g·cm^−3^). The effect of barite addition on the rheological properties of gel-forming suspensions and the viscoelasticity of the resultant supramolecular polymer gels was then systematically evaluated.

**Figure 3:**
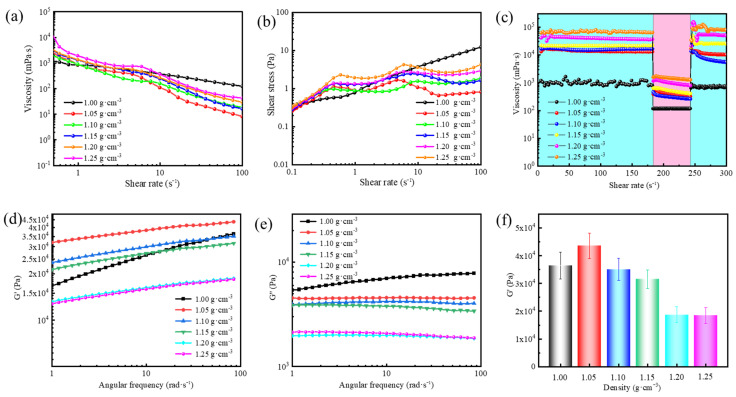
Effect of density on the rheological properties of gel-forming suspensions and the resultant supramolecular polymer gels: (**a**) viscosity versus shear rate of gel-forming suspensions, (**b**) shear stress versus shear rate of gel-forming suspensions, (**c**) thixotropic properties of gel-forming suspensions, Low shear is 10^−2^, and high shear is 10^1^. (**d**) G′ versus angular frequency of the resultant supramolecular polymer gels, (**e**) G″ versus angular frequency of the resultant supramolecular polymer gels, and (**f**) G′ at a fixed angular frequency of 84 rad/s.

Figure 3a illustrates the effect of different barite densities on the rheological properties of gel-forming suspensions. At a shear rate of 0.1 s^−1^, the suspension viscosity increased dramatically from 3413 mPa·s to 34,463 mPa·s as the density increased from 1.00 to 1.25 g·cm^−3^, showing a clear density dependence. In addition, the presence of barite enhanced the shear-thinning behavior, a non-Newtonian fluid characteristic that facilitates pumping operations during drilling. The shear stress trend in Figure 3b followed the same pattern as the viscosity behavior. Figure 3c displays the thixotropic properties of gel-forming suspensions, which enhanced with increasing barite density. The barite-filled gel-forming suspensions exhibited high viscosity at low shear rates and low viscosity at high shear rates, a characteristic that ensures effective retention of gel-forming suspension in fractures. Therefore, the presence of barite not only facilitated pumping operations but also improved the gel-forming suspension’s ability to stay in place and seal fractures effectively.

Furthermore, the viscoelasticity of the resultant supramolecular polymer gels was investigated. As shown in Figure 3d–f, the G′ increased as the density increased from 1.00 to 1.05 g·cm^−3^, followed by a gradual decrease from 1.05 to 1.25 g·cm^−3^. The increased G′ at a barite density of 1.05 g·cm^−3^ was attributed to the reinforcing effect of uniformly dispersed barite particles within the gel network. As more barite particles were included, the excess particles disrupted the homogeneity of the gel network, leading to stress concentration and a subsequent reduction in G′. The findings highlight the critical role of density in the performance of supramolecular polymer gels. Increasing density with an appropriate amount of barite enhances the viscosity, thixotropic behavior, and gel strength, which is beneficial for sealing fractures and maintaining gel integrity under pressure. However, excessive density can disrupt the gel network, causing stress concentration and reducing strength. Therefore, the balance between optimal barite concentration and gel performance is essential for ensuring effective sealing and flow control in drilling operations.

#### 2.2.3. pH

In addition to temperature and density, the chemical properties of the reservoir environment, especially the pH value, are also key factors affecting the performance of supramolecular gels. In the complex downhole geological environment, the pH of formation fluids exhibits significant spatial heterogeneity, which critically affects the gelation process and structural stability of supramolecular polymer gels. Herein, five pH gradients of 5, 6, 7, 8, and 9 were set using NaOH and HCl solutions as pH adjusters, and the effect of pH on the resultant supramolecular polymer gels was studied.

**Figure 4:**
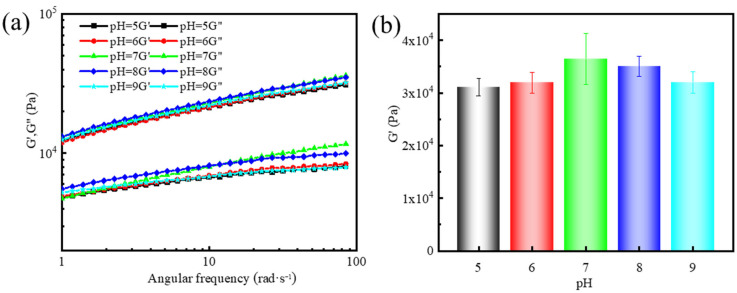
Effect of pH on the viscoelasticity of supramolecular polymer gels: (**a**) dynamic frequency sweep at a fixed strain of 10% and (**b**) G′ at a fixed angular frequency of 84 rad/s.

In the pH range of 5–9, gel-forming suspensions were also able to effectively form a three-dimensional gel network, with no significant differences in gelation time. The viscoelasticity of the supramolecular polymer gels was further analyzed, as shown in Figure 4a,b. The elastic moduli were 31,076 Pa, 31,948 Pa, 36,428 Pa, 35,064 Pa, and 31,957 Pa at pH values of 5, 6, 7, 8, and 9, respectively. These results indicate that the G′ reaches its maximum value at pH 7. Under this pH condition, the interactions between the active groups in the system reached an optimal state, with hydrogen bonding and electrostatic interactions playing a key role, resulting in a denser gel network structure and significantly enhanced gel strength. In contrast, a decrease in G′ was observed at pH 5 and 9, with reductions of 15% and 12%, respectively, compared to pH 7. This reduction is primarily attributed to the disruption of non-covalent interactions, such as hydrogen bonding and electrostatic interactions, that are critical for maintaining the integrity of the supramolecular gel network. At low pH (acidic conditions), excess H⁺ ions can protonate functional groups, weakening hydrogen bonds and electrostatic interactions. Conversely, at high pH (alkaline conditions), excess OH^−^ ions may deprotonate key functional groups, leading to increased electrostatic repulsion, which disrupts the balance of intermolecular interactions [40].

#### 2.2.4. Drilling Fluid Intrusion

During drilling operations, residual drilling fluid inevitably remains in the wellbore. When the gel-forming suspension is pumped downhole, it mixes with this residual fluid. The introduction of varying volume fractions of drilling fluid into the gel-forming system might not only alter the physicochemical properties of the gel-forming suspension, but also impact the strength of the resultant supramolecular polymer gel. To systematically evaluate the influence of drilling fluid on the properties of supramolecular polymer gels, five replacement ratios were selected for study: 10%, 20%, 30%, 40%, and 50%. The drilling fluid used in the experiment was composed of 4% bentonite and barite with a density of 1.05 g·cm^−3^.

**Figure 5:**
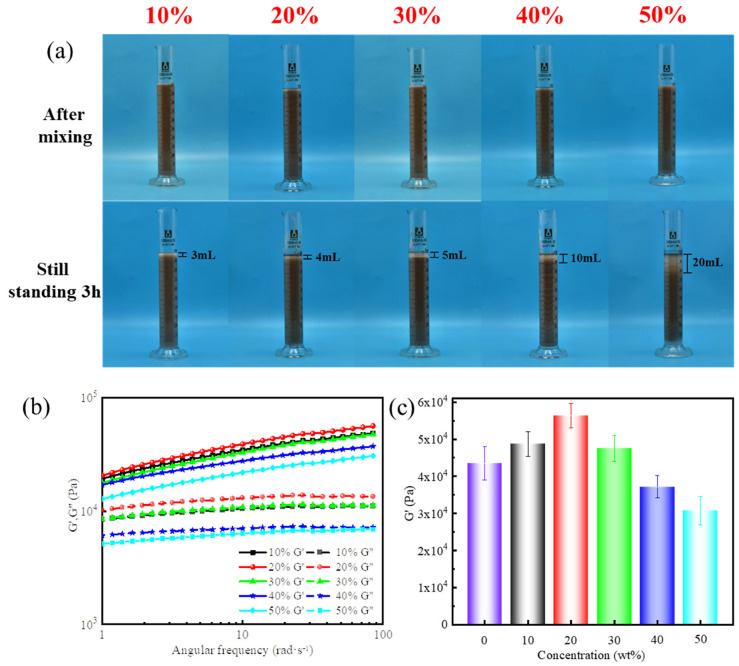
Effect of drilling fluid intrusion on the performance of gel-forming suspensions and supramolecular polymer gels: (**a**) appearance of the drilling fluid-filled gel-forming suspensions before and after standing, (**b**) dynamic frequency sweep at a fixed strain of 10%, and (**c**) G′ at a fixed angular frequency of 84 rad/s.

Figure 5a shows the appearance of the drilling fluid-filled gel-forming suspensions before and after standing for 3 h. The experimental results demonstrated a gradual increase in phase separation as the drilling fluid volume fraction increased from 0% to 50%. When the drilling fluid accounted for 10% of the system, the suspension maintained a homogeneous dispersion of solid particles after standing for 3 h. This stability was attributed to the synergistic hydrogen bonding and electrostatic interaction between the supramolecular polymer network and bentonite particles in drilling fluids. However, at a 50% volume fraction, the gel-forming suspension was largely diluted. Consequently, both the hydrogen bonding density and electrostatic interaction strength were significantly weakened, leading to strong sedimentation and phase separation.

Further dynamic rheological measurement on the resulting supramolecular polymer gels (Figure 5b,c) revealed that the G′ initially increased with increasing drilling fluid volume fraction, followed by a decrease as the volume fraction continued to rise. Particularly, as the drilling fluid fraction increased from 0 to 20%, bentonite particles in the drilling fluids acted as reinforcing additives together with CNFs and laponite, creating a more robust gel network. As a result, the G′ increased to 56,329 Pa, i.e., an increase of 23% compared with the control sample. However, at a 50% replacement ratio, the elastic modulus dropped to 30,753 Pa, yet the gel still retained sufficient strength to meet the fracture-blocking requirements.

#### 2.2.5. Saltwater Intrusion

When gel-forming suspension is injected into formation fractures, it is susceptible to dilution by various fluids present in the reservoir [38,41]. Among these, formation water, typically highly mineralized, is the most common and can impact the strength of resultant supramolecular polymer gels. Building on previous experiments involving drilling fluid intrusion, this section further investigated the effect of formation water intrusion on the performance of gel-forming suspension and resultant supramolecular polymer gels. To this end, a simulated formation water containing 70,000 mg/L Na⁺, 15,000 mg/L Ca^2+^, and 1000 mg/L Mg^2+^ was prepared, and then different amounts of simulated formation water (20%, 40%, 60%, 80%, and 100%, based on the volume of gel-forming suspension) were added into the gel-forming suspension containing 50% drilling fluid.

**Figure 6:**
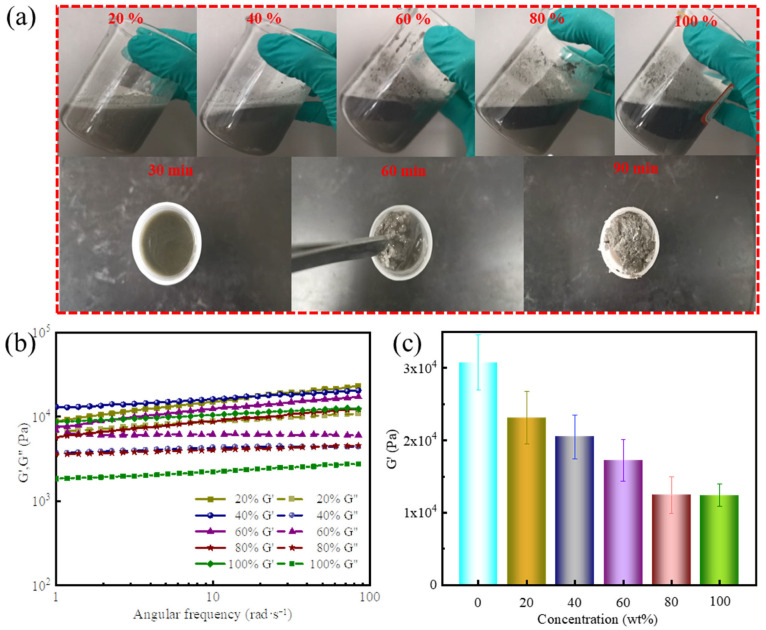
Effect of saltwater intrusion on the performance of gel-forming suspensions and supramolecular polymer gels: (**a**) appearance of gel-forming suspensions after mixing with 20%, 40%, 60%, 80%, and 100% saltwater (top), as well as the states of 100% saltwater-fill suspension at different gelation times; (**b**) dynamic frequency sweep at a fixed strain of 10%; (**c**) G′ at a fixed angular frequency of 84 rad/s.

Figure 6a shows the gel-forming suspension appearance following the addition of various proportions of saltwater, as well as the changes in gelation over time. As the formation water concentration increased, the suspension exhibited progressive stratification. This phenomenon is mainly attributed to the weakening of non-covalent interactions within the suspension, as the added salts disrupt the original equilibrium. However, even at 100% saltwater replacement, the system still achieved effective gelation.

Figure 6b,c show a decreasing trend in G′ with increasing saltwater content. At 100% formation water, the gel’s elastic modulus stabilized at 12,422 Pa. This reduction in strength is due to the salt-sensitive nature of the supramolecular polymer. Specifically, elevated concentrations of Na^+^, Ca^2+^, and Mg^2+^ promoted polymer chain coiling, and reduced the density of cross-linking sites within the gel network gel. These changes collectively weakened the gel lattice and decreased mechanical integrity. Overall, these results confirm that the developed supramolecular polymer gel exhibited tolerance to formation water contamination and retained sufficient strength for effective sealing, demonstrating its potential for reliable application in highly saline formations.

#### 2.2.6. Crude Oil Intrusion

During the drilling process, the drilling fluid system inevitably comes into direct contact with crude oil upon penetrating the reservoir. If excessive crude oil invades the gel-forming system, it might adversely affect its gel-forming behavior, reducing gel strength and sealing efficiency. Building on previous experiments involving drilling fluid and brine intrusion, this study further investigated the impact of varying crude oil intrusions on the performance of gel-forming suspension and resultant supramolecular polymer gels. Specifically, 2%, 4%, 6%, 8%, and 10% crude oil were added to the gel-forming suspension containing 50% drilling fluid and 100% saltwater.

**Figure 7:**
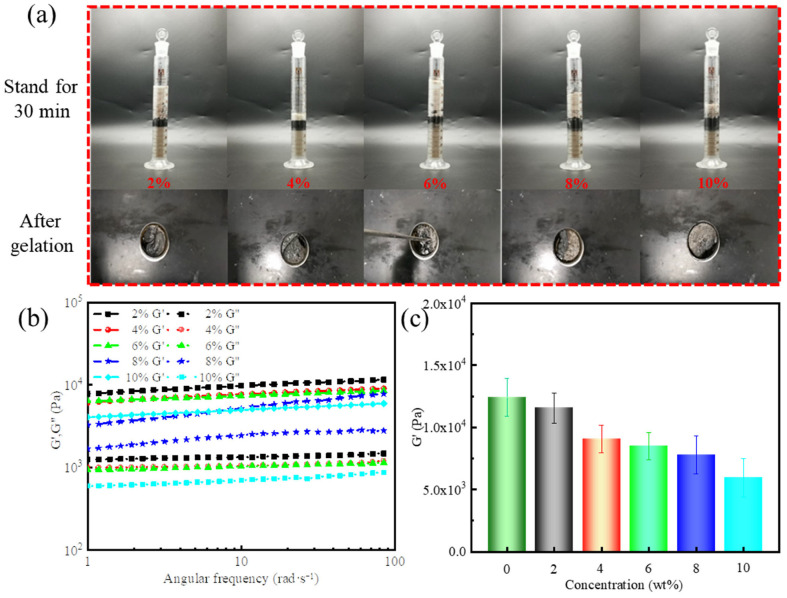
Effect of crude oil intrusion on the performance of gel-forming suspensions and supramolecular polymer gels: (**a**) appearance of crude oil-filled gel-forming suspensions after standing for 30 min (top) and gelation (down); (**b**) dynamic frequency sweep at a fixed strain of 10%; (**c**) G′ at a fixed angular frequency of 84 rad/s.

As shown in Figure 7a, distinct phase separation between the crude oil and gel-forming suspension became evident after 30 min, with the degree of separation increasing as the crude oil content rose from 2% to 10%. These results demonstrated that the gel-forming suspension exhibited a strong physical barrier capability against crude oil, and the interfacial layer formed can effectively hinder further crude oil penetration. In addition, gels were successfully formed in the presence of crude oil, maintaining structural integrity and the ability to seal reservoir fractures.

The effect of crude oil content on the viscoelasticity of supramolecular polymer gels is shown in Figure 7b,c. A consistent decrease in gel strength was observed with increasing crude oil concentration. Notably, even at 10% crude oil addition, the gel retained an elastic modulus of 5949 Pa. This weakening can be attributed to two key mechanisms [42,43]: (1) the non-polar hydrocarbon molecules in crude oil interfere with hydrogen bonding, electrostatic interactions, and cross-linking reactions via steric hindrance, reducing effective cross-linking between polymer chains; and (2) the increased presence of crude oil facilitates oil–water interface formation, which compromises the overall mechanical integrity of the gel.

### 2.3. Plugging Performance of Supramolecular Polymer Gel Under the Simulated Reservoir Conditions

The plugging strength of gels in pores and fractures is a key indicator of their effectiveness in lost circulation control. To accurately evaluate the plugging capability of gels in complex leakage channels, this study employed a steel column fracture core model and a sand-filled tube to simulate different formation conditions and systematically assess the plugging performance of the gel in varied media. The gel-forming suspension containing 15 wt% AM, 3 wt% AMPS, 2.6 wt% DVB, 5 wt% PVA, 0.30 wt% CNFs, and 3 wt% laponite, along with 50% drilling fluid (density of 1.05 g·cm^−3^), 100% saltwater, and 10% crude oil, was injected into the core fracture or sand-filled tube. Then, the temperature was set to a reservoir temperature of 120 °C. The plugging performance of the supramolecular polymer gel was systematically evaluated in two typical seepage models (i.e., pores and fractures) following the procedure described in Section 2.4.

**Figure 8:**
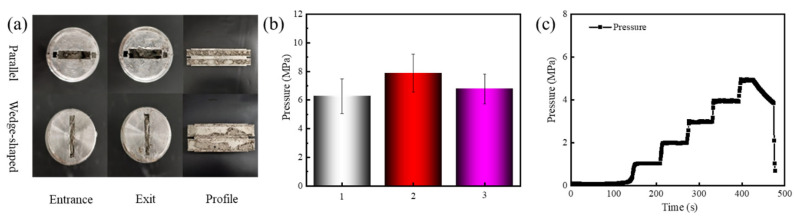
Plugging performance of supramolecular polymer gels: (**a**) macroscopic images after sealing with a supramolecular polymer gel system; (**b**) the maximum breakthrough pressure after sealing fractures of different widths (1: 2 mm × 2 mm parallel fracture, 2: 4 mm × 2 mm wedge-shaped fracture, and 3: 5 mm × 3 mm wedge-shaped fracture); and (**c**) the pressure-bearing curve of the sand-filled tube test.

For fractured formations, two fracture models with different geometries were selected to evaluate the maximum pressure-bearing capacity of the gel: (1) a 2 mm × 2 mm (entrance and exit widths) × 30 cm (length) parallel fracture; (2) a 4 mm × 2 mm × 30 cm wedge-shaped fracture; and (3) a 5 mm × 3 mm × 30 cm wedge-shaped fracture. The macroscopic images of the gel-filled fractures revealed that the gel system effectively achieved complete filling in both parallel and wedge-shaped steel column fractures (Figure 8a). Once gelled, the material remained uniformly distributed with good structural integrity, forming a strong barrier across different fracture sizes. The maximum breakthrough pressures recorded (Figure 8b) were 6.27 MPa for the 2 × 2 mm fracture, 7.89 MPa for the 4 × 2 mm wedge fracture, and 6.78 MPa for the 5 × 3 mm wedge fracture. In all cases, the gel demonstrated pressure-bearing capacities exceeding 6 MPa, confirming its strong sealing effectiveness in fractured formations.

For permeable formations, a porous medium was constructed using 40–100 mesh quartz sand. The plugging test was performed under a constant pressure injection regime, maintaining the initial pressure for 60 s before gradually increasing it. As shown in Figure 8c, with increasing injection pressure, the gel formed a dense and consolidated body within the sand pore structure, achieving complete blockage. Throughout the test, there was no observable fluid leakage or gel displacement by the replacement fluid, indicating that the gel material also exhibits excellent pressure-bearing and sealing performance in permeable formations.

### 2.4. Long-Term Stability Performance

The high-temperature environment underground poses a significant challenge to the long-term stability of gels, and their sustained performance directly impacts operational safety and efficiency. To systematically evaluate the long-term stability of the gel under high-temperature conditions, the following formulation was used: 15 wt% AM, 3 wt% AMPS, 2.6 wt% DVB, 5 wt% PVA, 0.30 wt% CNFs, and 3 wt% laponite, combined with 50% drilling fluid (1.05 g·cm^−3^), 100% saltwater, and 10% crude oil. The gel was aged at 120 °C and 140 °C for 7, 15, and 30 days, respectively, and its G′ values were measured after each aging cycle.

**Figure 9:**
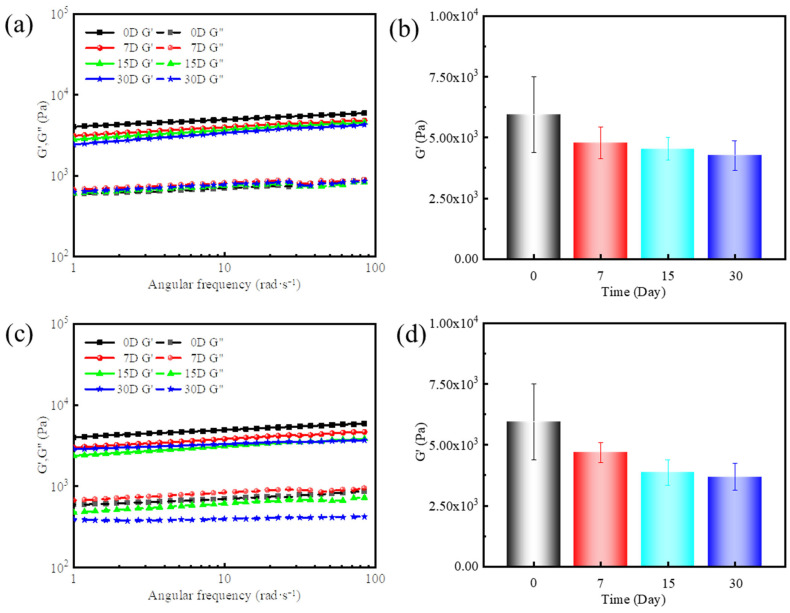
Stability of gel: (**a**) dynamic frequency sweep at a fixed strain of 10% at 120 °C, (**b**) G′ at a fixed angular frequency of 84 rad/s, (**c**) dynamic frequency sweep at a fixed strain of 10% at 140 °C, and (**d**) G′ at a fixed angular frequency of 84 rad/s.

Figure 9a–d show the changes in the gel’s elastic modulus over time under aging conditions of 120 °C and 140 °C. At 120 °C, the initial elastic modulus was 5948 Pa, decreasing to 4790 Pa, 4541 Pa, and 4266 Pa after 7, 15, and 30 days, respectively, corresponding to a cumulative decay rate of 28% over 30 days. At 140 °C, the elastic modulus values were 4685 Pa, 3866 Pa, and 3689 Pa after 7, 15, and 30 days, with a cumulative decay rate of 38%. These results indicate that although the gel’s elastic modulus decreases with prolonged high-temperature aging, the strength loss remains relatively limited. After 30 days, the gel retains 60–70% of its initial strength, demonstrating excellent long-term thermal stability for high-temperature applications.

### 2.5. Biodegradability

In the study of supramolecular polymer gels for sealing fractures in geological formations, evaluating their biodegradability is essential to ensuring their environmental friendliness. Chemical oxygen demand (COD) and biochemical oxygen demand (BOD) are important indicators used to assess the degree of organic pollution in water and its biodegradability. By measuring the COD and BOD of the gel-forming suspension, we can evaluate its potential environmental impact and determine whether it complies with environmental protection standards. In this study, the COD of the gel-forming suspension was measured at 58,800 mg/L, and the BOD at 31,077 mg/L, yielding a BOD/COD ratio of approximately 0.53, which is much higher than the biodegradability threshold of 0.25. This indicates that most of the organic matter in the solution can be efficiently metabolized by microorganisms, thereby meeting environmental regulations regarding biotoxicity and ecological impact. The suspension demonstrates good environmental compatibility and can be safely applied in geological formation sealing.

## 3. Conclusions

In this study, the effects of reservoir conditions on the performance of supramolecular polymer gel-based lost circulation materials were systematically investigated, and the following main conclusions were drawn:

(1) The optimal formulation of the supramolecular polymer gel was determined through orthogonal experiments. This formulation consisted of 15 wt% AM, 3 wt% AMPS, 2.6 wt% DVB, and 2.6 wt% PVA. The addition of 0.30 wt% CNFs and 3 wt% laponite significantly enhanced the gel strength and promoted the formation of a highly cross-linked three-dimensional network structure.

(2) Experimental studies demonstrated that temperature significantly affected both gelation time and strength, with elevated temperatures accelerating gelation while reducing mechanical strength. Increased density enhanced the initial viscosity and thixotropic properties, with all formulations exhibiting characteristic shear-thinning behavior. The gel strength demonstrated a non-monotonic dependence on density, achieving maximum values at intermediate densities. A robust three-dimensional network structure developed within a specific pH range, with the highest strength observed at a neutral pH. Moderate drilling fluid contamination enhanced gel strength, whereas formation water and crude oil intrusion exhibited detrimental effects.

(3) The gel exhibited excellent plugging ability across fractures of various widths (2–5 mm) and in permeable formations (40–100 mesh). The maximum breakthrough pressures exceeded 6 MPa for all fracture types. In permeable media simulations, the gel formed a dense, solid body that completely blocked fluid flow, with no noticeable leakage.

Therefore, the developed supramolecular polymer gel-based lost circulation material demonstrates broad adaptability to complex reservoir conditions. While the optimized formulation demonstrates excellent gel strength and plugging performance, the relatively high content of monomers and functional additives may pose cost challenges for field-scale applications. Future work should focus on improving cost-efficiency by partially replacing functional components with inert fillers (e.g., silica and CaCO_3_), which can reduce raw material costs without significantly compromising gel performance. Field testing will also be essential to verify the gel’s mechanical and rheological properties under practical operational conditions. This study lays a strong foundation for the use of supramolecular polymer gels in complex formation environments, offering a promising solution to lost circulation challenges in oil and gas development.

## 4. Materials and Methods

### 4.1. Chemicals

The chemicals and materials, including polyvinyl alcohol (PVA), 2-acrylamido-2-methylpropanesulfonic acid (AMPS), acrylamide (AM, with a purity exceeding 98%), ammonium persulfate (APS; resin-coated), and divinylbenzene (DVB), were all purchased from Aladdin Chemical Co., located in Shanghai, China. TEMPO-oxidized cellulose nanofibers (CNFs) with a solid content of 2.6 wt% were supplied by Shenquan Group Co., Ltd., based in Jinan, China. Llaponite (Li_2_Mg_2_O_9_Si_3_), with an average diameter ranging from 25 to 30 nm and a thickness of 1 nm, was generously provided by Lanabai Pharmaceutical Chemical Co. in Wuhan, China.

### 4.2. Synthesis of Supramolecular Polymer Gels

We synthesized supramolecular polymer gels through free radical polymerization by first dissolving PVA in deionized water at 95 °C with continuous stirring. We then sequentially added the reaction components (e.g., AM, AMPS, DVB, APS, CNFs, and laponite) to the solution under thorough mixing. Among these, the concentration of nanomaterials was selected based on two factors. On the one hand, the dosage range of CNFs and laponite was mainly based on our previous work and literature research [8]. On the other hand, the selected concentration was based on its known threshold for forming a percolated structure that contributes to the gel-like behavior of the suspension [44]. The resulting gel-forming suspension underwent polymerization at controlled temperatures (40–140 °C) to form the final network structure.

### 4.3. Characterization of Supramolecular Polymer Gels

The appearance of the supramolecular polymer gels was recorded using a digital camera.

The dynamic frequency sweep of the supramolecular polymer gels was performed using a HAAKE rheometer (HAAKE MARS 60, Karlsruhe, Germany) at 25 °C, with a fixed strain of 10% over a frequency range of 0.5–100 rad/s.

The plugging capacity of supramolecular polymer gels in fractures was evaluated using a high-temperature, high-pressure plugging apparatus (High Temperature High Pressure Carcassing Device, Qingdao, China) under simulated reservoir conditions. First, the gel-forming suspension was prepared according to the optimized formulation and injected into the drilling fluid kettle to assemble the experimental setup. Next, the fractured core or sand-filled tube was placed in the core holder and subjected to confining pressure to simulate geological stress conditions. An advection pump was used to inject the gel-forming suspension into the core fracture or sand-filled tube. Once the filtrate flow at the outlet stabilized, the outlet valve was promptly closed to maintain system integrity. The core holder was then heated to the target formation temperature to simulate in situ gelation conditions. After complete gel solidification within the fracture or sand-filled tube, the outlet valve was reopened, and water was injected to test sealing efficiency. A pressure sensor continuously recorded the pressure in the reaction chamber, and the maximum breakthrough pressure was used as the key indicator of the gel’s sealing and pressure-bearing capacity.

## Figures and Tables

**Table 1:** Orthogonal factor level table.

Level	Factor				
	A: AM/wt%	B: AMPS/wt%	C: DVB/wt%	D: PVA/wt%	E: APS/wt%
1	5	1	1.3	5	0.065
2	10	2	2.6	7	0.065
3	15	3	3.9	9	0.065

**Table 2:** Orthogonal experiment of supramolecular polymer gel.

Number	A	B	C	D	E
1	1	1	1	1	1
2	1	2	2	2	1
3	1	3	3	3	1
4	2	1	2	3	1
5	2	2	3	1	1
6	2	3	1	2	1
7	3	1	3	2	1
8	3	2	1	3	1
9	3	3	2	1	1
K1	1535	2798	3669	4687	
K2	3889	3049	4249	3155	
K3	5223	4800	2729	2806	
R	3688	2002	1520	1881	
optimal choice	A_3_	B_3_	C_2_	D_1_	
priority	A > B > D > C				

## Data Availability

The original contributions presented in this study are included in the article. Further inquiries can be directed to the corresponding author.
